# Stigma, epistemic injustice, and “looked after children”: The need for a new language

**DOI:** 10.1111/jep.13700

**Published:** 2022-05-22

**Authors:** Danielle Fieller, Michael Loughlin

**Affiliations:** ^1^ Nottinghamshire Healthcare NHS Foundation Trust Nottingham UK; ^2^ School of Biomedical Sciences University of West London London UK

**Keywords:** abuse, epistemic injustice, Fricker, “looked after children”, safeguarding, stigma

## Abstract

This article examines the processes that contribute to the stigmatization of a group of people typically identified as “children in care” or “looked after children.” In particular, we will look at the ways that we (adults, professionals, and carers) interact with these children, based on their status as both children and members of a socially marginalized and disadvantaged group, and how these modes of interaction can inhibit dialogue—a dialogue that is needed if we are to base our conceptions regarding the needs of these children on a more accurate understanding of their experiences and perspective. The problem is particularly challenging because the very terminology we use in the care community to identify this group is a product of the damaging preconceptions that have affected our interactions with its members and, we argue, it serves to reinforce those preconceptions. Using Fricker's work on epistemic injustice, in conjunction with evidence regarding how accusations of abuse and neglect of these children have been addressed in numerous cases, we illustrate the problems we have in hearing the voices of members of this group and the harmful effects this has on their own ability to understand and articulate their experiences. These problems represent “barriers to disclosure” that need to be surmounted if we are to establish a more inclusive dialogue. Currently, dialogue between these children and those of us charged to “look after” them is too often characterized by a lack of trust: not only in terms of the children feeling that their word is not taken seriously, that their claims are not likely to be believed, but also in their feeling that they cannot trust those to whom they might disclose abuse or neglect. The goals of the paper are modest in that we aim simply to open up the debate on how to meet this epistemic challenge, noting that there are specific problems that extend beyond those already identified for hearing the voices of other victims of epistemic injustice. Explicitly recognizing the nature and extent of the problem still leaves us a long way from its solution, but it is a crucial start.

## 1 INTRODUCTION

Authors since Goffman^1^ have recognized the importance of understanding the interactional production of stigma, and the need to identify and address discrimination, degradation, and discreditation in a wide range of contexts—where those who suffer these social processes and associated attitudes are often members of minority and vulnerable groups. Fricker's important work on epistemic injustice^2^ has facilitated a crucial focus on discrimination and the discreditation of individuals specifically regarding their status as *knowers*, reducing their credibility as reporters of events and experiences, and also inhibiting their own ability to make sense of those events and experiences. Her analysis provides an opportunity to reduce stigma and combat injustice via the development and promotion of languages that enable us to reconceptualize harmful social interactions, to render previously distorted or invisible injustices transparent. Doing so represents the necessary first step in any process to rectify these injustices.

This paper looks specifically at the group of people typically identified as “children in care” or “looked after children.” We will use this terminology (frequently abbreviated to “LAC”) throughout the discussion, because of its dominance in practice, particularly in professional, local authority, and department of health discourse.^3^, ^4^, ^5^ However, one of our conclusions is that these terms will themselves need to be replaced as the debate progresses, if those of us charged with caring for these children are to hear their voices and treat them appropriately. The goals of this paper are modest in that we are calling explicitly for the beginning of a debate aimed at revising our language and conceptual framework in the care community, considering some of the challenges this process will face and sketching some ideas regarding how to meet those challenges—sketches we very much hope others will join us in amending and completing.

## 2 DIALOGUE AND EXCLUSION: THE SPECIAL CHALLENGES PRESENTED BY LOOKED AFTER CHILDREN

Children in care are particularly vulnerable to exclusion and face discrimination from their peers and in the community, by virtue of being looked after.^6^ This injustice diminishes their capacity as a knower resulting in a testimonial depreciation, possibly preventing future speaking out.^7^ Fricker^2^ argues that it is important to develop a language to recognize explicitly such forms of injustice, as the first step to seriously combatting them. Drawing on feminist theory, Fricker talks about the historic under‐representation of women in the development of important dialogue. Fricker notes that, before the development of the language of “sexual harassment”, women may have struggled to make sense of their experiences and feelings in certain contexts. A woman who felt uncomfortable in the workplace could be seen as humourless for failing to “play along” with “office banter” of a sexual nature. She might be encouraged to see herself as the problem, diagnosing her own inability to join in the “fun” as indicative of a psychological problem on her part—that she is prudish, sexually repressed, or otherwise inhibited. In extreme cases, a woman could feel under pressure to demonstrate her “normality” by laughing and encouraging behaviour following such banter, eventually being led into nonconsensual sexual behaviour and relationships on the grounds that she had “led on” a male colleague or acquaintance.

But when a new language developed, incorporating the terminology of “sexual harassment,” women could come to see their situations, including their own feelings of discomfort in particular social contexts, in different ways. The failure of the “banter” to amuse her no longer indicates that she is the problem. Conceptually, the language of “sexual harassment” redraws ethical boundaries, making certain types of comment and behaviour an unwarranted intrusion or “crossing of a line,” and feelings of discomfort can then be seen as natural reactions, legitimate concerns generated by an encroachment of the woman's autonomy. Women can make sense of their feelings of discomfort, rather than seeing them as a psychological problem. This conceptual shift has significant practical implications, reinforcing their ability to affirm their right not to take part in social practices they regard as degrading or threatening.

The language of epistemic injustice can help us to understand social processes that predate its own development. The English philosopher Mary Astell (1666–1731) revealed the existence of a form of epistemic injustice she called “bad custom.” Astell identified that this is not other people failing to take women seriously because they're women. It is something that makes women underestimate their own credibility. This is an incredibly powerful social structure that continues to exist in the 21st century. Through bad custom, it becomes difficult to see how a woman would be able to trust her own knowledge if someone with “greater intellectual ability” (a man) tells her that she is mistaken.^8^ If bad custom can have such a long‐standing effect on adult women, it should come as no surprise that a child can be led to question her own knowledge when contradicted by an adult.

According to Fricker, there are two forms of epistemic injustice: hermeneutical and testimonial.^2^ Testimonial injustice arises when an individual is not recognized as a credible informant because of a bias on the hearer's part. This then leads to the discrediting of another in “their capacity as a knower.”^9^ Testimonial injustice is particularly evident in the treatment of members of marginalized and disadvantaged groups.^10^ Hermeneutics concerns how we understand and interpret both words and experiences. Hermeneutic injustice occurs when people's experiences are not adequately understood or expressed (by others or indeed themselves) because of what Fricker calls “a deficit in our shared tools of social interpretation (the collective hermeneutical resource).” Both of these forms of injustice are relevant to the discussion of the group identified in the care community as “LAC.”

As the evidence cited in the next section indicates, there is an urgent need to address testimonial injustice relating to looked after children. As practitioners, we need to examine and question the preconceptions that affect our judgement when considering claims made by these children. Only by doing so can we create an environment in which children feel able to disclose abuse they have experienced. Clearly, children and members of socially disadvantaged minority groups are not leaders in developing linguistic dialogue. Children in care fall into both categories, and as a result are not supported in finding the right way to conceptualise or articulate the damage being done to them. So hermeneutic injustice is also relevant. To change attitudes and practice, to prevent the epistemic injustice affecting them, and to reduce the identified barriers to disclosure, we must develop a language to express forms of injustice previously not identified.

There is already an epistemic challenge in hearing the voices of children.^11^ Is it possible that looked after children may fall outside of Fricker's discussion of epistemic injustice because they are less likely to share information?^12^ Fricker's illustrations related to the historic exclusion of women from the development of our shared tools of social interpretation.^2^ Others have discussed the exclusion of members of oppressed racial groups from the development of dominant languages that serve to understand and characterize our social relationships. As Lagewaard^13^ observes, “epistemic resources are developed and shared to reflect the experiences of communities.” However, in the context of oppression, the members of some communities “might be epistemically left out of the process of developing or sharing these resources.” He notes that those communities may well “form their own resources, reflecting their experiences,” but that these resources will not be “shared on a communal level,” resulting in a lack of shared vocabulary.

There are additional problems in including children, the socially marginalized, and (as Dohmen^12^ notes) people with mental health problems in the hermeneutic process—involving them in discussions about how to interpret shared social experiences. Clearly, we need to distinguish between harmful stereotypes and legitimate differences in our approach to engaging in dialogue with children, on the one hand, and members of the communities that are the focus of Lagewaard, on the other. Whilst the theoretical framework of epistemic injustice has been applied in other areas, in mental health care, it is arguably still underused.^14^ Looked after children are already disadvantaged educationally, financially, and emotionally. They are often on the receiving end of stigma, epistemic injustice, and social isolation. Unlike members of the communities Lagewaard considers, these children may be unable, unassisted, to “form their own” epistemic resources, by developing languages shared by other members of their social group to characterise their specific understanding of their own experiences. So, there are problems of a different nature from those which have prevented women and members of racially oppressed groups from sharing such hermeneutic resources with the broader community.

## 3 REPORTING ABUSE—BARRIERS TO DISCLOSURE

Arguably, a child is afforded less testimonial credibility than warranted, particularly if a child relies on an adult playing a central role in the development of testimony.^15^ Can a testimony be successful if the child is experiencing abuse or neglect at the hands of those responsible for ensuring their well‐being?^16^ Even when the adult carer is not an abuser, the credibility of the child's testimony can be undermined by the attitudes of the carers. Utting's research^17^ noted that children and young people placed in residential care may be at risk of abuse by their peers. Some of those who had reported sexual abuse by peers complained that this was not taken seriously, because staff sometimes viewed this as consensual sexual activity.^18^

Such children are legally entitled to report abuse, as are other children, but we know that legal rights alone are insufficient if not fully supported by appropriate social processes. Children can complain to a UK court if their rights have been violated, and if the claim is rejected, take their claim to the European Court of Human Rights.^19^ That said, how many of us have ever met a child who would know how to action this? (It seems likely that many adults would not know how to action this, either.) But if a child did want to complain to the ECHR would the adults listen? At the age of eight one of us (Fieller) quoted the children's act (1989) to a year four teacher. She did not really understand the section of the act being quoted but knew it held value. The teacher was bewildered and somewhat annoyed. Why? Did the question challenge the teacher's position as an adult who ‘knows better'? Are children being implicitly taught to doubt their own ability to contribute to discourse that directly concerns them, in much the same way that the women discussed by Astell learned the impropriety of challenging men who obviously “know better”?

Children learn from example—they learn to swear, smoke, fight, and to reproduce. Imagine a world where learning about legislation, human rights, feminism, immigration, and democracy is part of the national curriculum. Adults have a responsibility to arm children with knowledge. Making this the “norm” would be a powerful way to protect children, but also to empower them with a means of emotional self‐defence. We also have a responsibility to educate ourselves, if we are to create the conditions to enable children in care to reach their full potential.^20^ Children in care are a particularly vulnerable group, experiencing extreme disadvantage compared with their peers.^21^ Some children are afforded a privileged care pathway, while others are not. Understanding individual barriers will enable health and social care professionals to act accordingly at the pivotal point of disclosure, without controlling how the abuse narrative is handled. A child may not wish to disclose at all; however, the decision to disclose is often made from fear, and the fear of the unknown remains after the disclosure has been made. Therefore, informing and consulting the child throughout the disclosure process is crucial.^22^ The example we give them will inform their developing conception of the social world and their self‐conception, affecting their ability to trust, share information, and express their feelings.

Allnock and Miller emphasise that children may make several disclosures before any action is taken.^23^ This often results in an inadequate safeguarding response preventing the child from being effectively protected.^22^ Kelly^9^ argues that the discrediting of an individual testimony leads to harm individually and collectively, often resulting in the mental health needs of the “LAC” being institutionally ignored. Such children are often disregarded, or not considered to meet the criteria for legal intervention.^9^

Being listened to, taken seriously, treated as a credible source of information—these things are central to a child's testimony because they need to have the confidence that they will be believed to make the disclosure: often, they are the sole witness to the abuse.^24^ A child's testimony must be considered crucial in determining safe and secure care and treatment. Adults have an ethical responsibility to listen to children, so learning to listen is the first obstacle to be overcome. Next, we must develop a language that is less clinical and more person‐centred. In this context, being more “person‐centred” means taking into account the specific experiences and perspectives of the children, treating them as a form of evidence that is quite distinct from, but no less valuable than, clinical evidence.^25^ It may be evidence that is difficult to access and requires careful interpretation. But methodologically, the use of narrative or “anecdotal” evidence cannot be dismissed or regarded as inherently “lower grade” than other forms of evidence.^26^ As Henry^27^ notes, understanding the perspectives of one's fellow human beings involves a form of engagement fundamentally different from the kind of reasoning involved in solving scientific problems. However, a review of the literature in this area reveals that the competency and skills required to assess a child's credibility at interview, and how practice impacts upon credibility, have been overlooked.^28^

Gallagher^29^ has published research detailing the disturbing extent of institutional abuse. Among practitioners, policymakers, and members of the public, he identifies this as a focus of major concern. Despite this, knowledge about it remains limited. A serious case review entitled “The sexual abuse of children in a foster home”^30^ investigated the historical sexual abuse of looked after children between 1999 and 2008 in Hackney, London. One of the abusers was an approved foster carer who remained in this role almost two decades. The overview report identified that the young people who were sexually abused did not disclose what had occurred until many years later. When they did, some chose to confide in their trusted friends, rather than report what happened to professionals. This is a common theme and a massive barrier to disclosure. The same report indicates that there was an earlier opportunity to prevent the abuse of some of the children. Despite this, the local police failed to investigate this allegation but kept the information as intelligence for possible future use.^30^ This is very serious breach of responsibility to protect the public and an illustration as to why these children are distrusting of the very same adults who a have a duty to keep them safe. There is an underlying assumption that children who are looked after are not at risk from their carers. It is vital that individual testimony is listened to by professionals with a need to be open minded. The possibility that a child can be harmed by a person who has a professional role in the child's life must not be dismissed.^30^ Davies and Wright^31^ conducted a literature review of children's views of mental health services. The data revealed that the interaction between children and staff is influenced by previous damaging interactions with adults. This finding was particularly prominent in children in care.

Biehal, Cusworth, and Wade reported on “Keeping children safe: allegations concerning the abuse or neglect of children in care.”^16^ The study described the nature of confirmed abuse or neglect for 118 children. They found that “all forms of maltreatment were evident,” including physical abuse (in 37% of cases), emotional abuse (30%), sexual abuse (11%), and neglect (17%). In addition, 15 cases were reported to concern “poor standards of care falling short of actual abuse.”

Significantly, the authors also reported on the difficult dilemmas professionals are often presented with when deciding on an appropriate course of action following an allegation or a disclosure. The disruption children are faced with when they are removed from placement is often unnecessary. Nonetheless, the alternative poses a greater risk of further exposure to harm. Similarly, a knee‐jerk reaction to an unsubstantiated allegation or disclosure can expose foster carers to unwarranted scrutiny and suspicion.

## 4 SURMOUNTING THE BARRIERS

The centre of expertise on child sexual abuse argues that sexual abuse in residential care in England is underreported.^32^ The report suggests that this is likely due to children fearing reprisal. Arguably, there are signs of sexual abuse in children before a verbal disclosure is made.^33^ Challenging behaviour is an example of this. Carers, and health and social care professionals have a responsibility to be alert to this risk and to act accordingly. Children who have been sex trafficked may continue to face an external threat from the trafficker. Some local authorities have concerns that a child being placed in a residential setting can escalate this risk.^34^ Consistency of care is an effective way to manage the risk. If a looked‐after child has a key worker with whom she can build a trusting and meaningful relationship, this helps her to feel secure. Those carers/staff need specific training to support a child's individual needs.

A combination of organisational processes, poor service user/professional relationship, and extreme service pressures contribute to superficial child protection.^35^ It is well documented that the aforesaid is directly linked to child abuse cases which have resulted in either the serious harm or death of a child.^36^ The phenomenon is now known as the ‘invisible child'. A well‐known example is Lord Laming's enquiry^37^ into the sustained torture, abuse, and murder of 8‐year‐old Victoria Climbié—who was subject to a kinship placement and subsequently killed by a family member. During Victoria's short life in Britain, she was known to four local authorities, two child protection police teams, two hospitals, and an NSPCC centre. Lord Laming's enquiry heard that many of the councils were understaffed, underfunded, and poorly managed. At the trial following Victoria's death, the judge described the people in charge of her case as “blindingly incompetent.”^38^ While it is hard to dispute this assessment, it would be a mistake to see this as a case where the child was just unlucky to have a particularly incompetent group of people in charge of keeping her safe. We need to recognize that there is an underlying cause for this sort of widespread “incompetence,” and that addressing it is the way to ensure greater competence in future.

In 2014, an NSPCC report found that 8 local authorities had more than two‐and‐a‐half substantiated allegations per 100 looked after children, and 43% of the foster carers involved had previously been subject to allegations.^16^ This demonstrates a high level of weakness in safeguarding policies and procedures. It also indicates that the system is providing an inadequate service to children in care.

In 2020, the BBC reported that Scotland introduced a new law to protect children giving evidence in criminal cases.^39^ Child witnesses in the most serious crimes are no longer asked to give evidence in court. The Barnahus system (a Scandinavian term meaning “child's house”) was first developed in the United States before being adopted in Iceland in the late 1990s. The principles and standards of the new system are child‐centred with an MDT approach to ensure that the rights and well‐being of children are always respected. It draws on the Scandinavian experience by reducing the harm and trauma often caused by the number of interviews a child must go through if they are a witness or victim. This act means more children will now be able to give prerecorded evidence in an environment more suitable to their needs, the challenge being how to strike the right balance between representation and safeguarding the child's right to be heard.^40^

Erens et al.^41^ argue that there are cases in which questionable interviewing techniques appear to have led to false memories of abuse in children. So, for this process to be effective, the professionals interviewing the children must have knowledge of children's memory function, together with excellent forensic interviewing skills because they are vital in child abuse investigation cases.^41^ These findings are worrying because health and social care professionals are often the first people to interview children making an abuse disclosure. If the child being interviewed holds an inaccurate memory belief then this fact, coupled with inadequate interviewing methods, could result in errors. Empirically based approaches for interviewing children are crucial in preventing this.^41^

Research indicates that child protection systems in developed nations, including Australia, Canada, and Britain, are outdated and reactive.^42^ For a system to work it relies on safeguarding being everyone's business. But that also relies on a case‐by‐case approach and for maltreatment to have already occurred. Even then a safeguarding concern must meet a threshold criterion for children's social care involvement. This is arguably a rigid approach as identified in Nottinghamshire Children's Services “Pathway to Provision” handbook.^43^ It supports practitioners to identify an individual level of need and to enable the most appropriate referrals to access provision. Yet, the children living in underprivileged socioeconomic circumstances will automatically face a disadvantage by virtue of the economic, material, and psychosocial conditions in which they grow up.^44^ Epistemic injustice applies particularly to disadvantaged children with difficult experiences. Do adults inadvertently silence children with discursive positioning in the context of child protection policy? Knezevic^7^ argues that by raising questions about epistemic injustice we are recognizing it is imperative to grant children recognition as knowledgeable agents who are capable of moral reasoning. Adults have a responsibility to empower children to have the loudest possible voice and the opportunity to influence decisions about their lives. This responsibility is especially significant within the context of children having to participate in welfare investigations and assessments.

Children in care who have been sexually abused are not a homogeneous group and their trauma is expressed in various ways. The individual's experience of child sexual abuse requires an adult carer and professional to understand the subjective needs of the child. It is essential to understand the different risks presented by the child's disability, gender, ethnic, cultural, religious, and linguistic needs to protect them. For example, it is expected that girls will mask their distress. This is often in the form of social withdrawal, anxiety, depression, and experiencing somatic symptoms. In contrast, research suggests that boys are more likely to externalize their distress with anger, suicide attempts, and engaging in risky criminal/sexual behaviours.^45^ This often leads to health and social care professionals misinterpreting the signs of sexual abuse due to a lack of training. Disabled children are more likely to experience institutional abuse due to their care setting. This is because of their physical dependency on others for personal care. Coupled with isolation and reduced autonomy this often places them at a higher vulnerability. Disabled children often have a reduced means of communication. However, their distress and trauma have the same presentation as nondisabled children.^46^ Equally, the appropriate education and training is vital for adults caring for black, Asian, and minority ethnic children. They are more likely to be looked after and less likely to be adopted.^47^ Therefore, to support learning about their ethnicity, identity, and culture, adults must develop strategies for resisting racism.

A prominent theme of young people who experience abuse is they do not talk about it.^48^ This does not mean they have not attempted to disclose the abuse in some way—rather that the abuse has not been “heard” or acted upon.^49^ The NSPCC completed a report in 2014 that describes the childhood experiences of abuse of 60 young men and women and how they disclosed abuse and sought help.^16^ The report identified that many of the young people interviewed described being unable to understand or articulate their feelings at the time of the abuse, despite knowing something was wrong. This was alongside feeling threatened and afraid of the abuser, so they remained silent. Others described feeling ashamed and embarrassed by their experiences, or afraid of being accused of lying.

Policymakers and people working with children must familiarize themselves with the available research to support better identification of abuse by adults. In turn, this will reduce the barriers to disclosure and improve the experience of disclosure for young people.^50^ Health and social care professionals must change their practice to prevent childhood abuse. An important finding of the report was that some young people felt unable to disclose their abuse but felt they would have liked someone to notice or ask them. This can work as a powerful motivator.^51^ As part of a mental health assessment process, exploring the topic of historical or current abuse is imperative in a thorough risk assessment. Children and young people are more likely to make a disclosure if an adult takes notice of their struggles and asks them. This can be through direct questions or building a therapeutic relationship over time.^52^ Not being noticed, being asked, or being heard is a consistent key finding in disclosure delays or failures. Even if a young person is not ready to make a disclosure, there is evidence that being asked provided a safe and supportive pathway to seek help later when they were ready.^23^ It is not recommended or good practice for a young person to be required to meet with professionals and the alleged perpetrator of the abuse following a disclosure. This is likely to be met with a young person withdrawing the disclosure and continuing to suffer abuse.^53^

Disclosures that result in a positive experience for young people have the prominent theme of being “believed,” followed by emotional support to help the young person through the process because disclosure is a journey. Consistency of care here is imperative for relational security. The young person must have the opportunity to build a rapport with a key person throughout the process. Without this, they are unlikely to continue through an emotionally distressing situation. They must have someone they can turn to.^49^

## 5 CHANGING OUR LANGUAGE

Looked after children are arguably one of society's most vulnerable groups. The way we interact with children is different to the way we interact with adults, and the way we characterize those children frames the interaction—language matters. Health and social care professionals must recognize there is a certain stigma attached to the language we use and for children this can often feel embarrassing. The terms “looked‐after child” and “child in care” are glib.^1^ This language unfairly injures their right to be viewed and treated like any other child. Is this having an adverse effect on the children we care for? Most certainly, the children and young people we care for should be referred to by their name. Collectively, however, using alternatives to the words “LAC” and “children in care” would be a good start to making care more person‐centred. As the sources cited in the preceding sections of this paper indicate, this language has become too closely associated with harmful stereotypes and an automatic tendency to disregard or dismiss statements made by members of the group it characterizes. The epistemic challenge of hearing the child's voice begins with challenging the care system jargon.

As health and social care professionals, we are complicit in a system that functions like a “corporate parent.” In fact, this is a term widely used in both health and social care, meaning “the collective responsibility of the council, elected members, employees, and partner agencies, for providing the best possible care and safeguarding for the children who are looked after by us.”^54^ This is the language we use in front of children in care. Although without malice, it is also without consideration of the consequences for their self‐image. These are children who already feel scared, stigmatized, and confused. Put simply, this is the discrimination of children from a marginalized group. Given its associations, if we applied such language to race or gender it would rightly be illegal. Certainly, if we used the fact that a person was from a particular racial group to infer that their claims needed to be treated with greater caution than the claims of others, we would be instantly recognized as being guilty of unacceptable prejudice.^2^ Looked after children have been omitted from the general equality debate by both individuals and institutions.^6^ This discrimination is so deeply imbedded within the care system that the Local Authority is the only parent in England that never faces the threat of legal action for failing to protect children.

To be a victim of abuse as a child is much more than trauma. It is a misplaced childhood, fear, anxiety and to feel different to your peers. For the child to face epistemic injustice because of this means we have failed not only as professionals but also as a society that is trusted to protect children. In the UK, almost 50,000 children are now in the care of councils whose services are regarded by Ofsted as either “inadequate” or “requiring improvement.”^55^ The disadvantage faced by children in care hugely impacts their life chances. In England, only 13.2% of children in care obtain five A‐C GCSEs, compared with 57.9% of their peers who are not in care, and a quarter of girls in care are pregnant before they're 18.^56^ This is a silent crisis that is getting worse, not better. Children in care have no political capital. So, they are relatively risk‐free for political institutions to overlook, given that there is almost no possibility of political consequence in the short to medium term. Yet these are precisely the vulnerable groups that our representatives are supposed to advocate for: the ones who can't speak for themselves. We should perhaps stop thinking of the children as “damaged” and think instead of the system as “damaged”—a system that does not know a child individually, but as a statistic requiring a legal intervention. Thus, because of their experiences both before and during care, these children are likely to go on to develop profound and long‐lasting mental health problems in adulthood.^57^

Fricker is concerned with the credibility deficit that follows a person through life in a variety of contexts. This stereotype is defined as: “a widely held disparaging association between a social group and one or more attributes, where this association embodies a generalization that displays some (typically, epistemically culpable) resistance to counter‐evidence owing to an ethically bad affective investment.”^2^ Fricker uses a scenario from *To Kill a Mockingbird* to illustrate epistemic injustice. Tom Robinson is a black man who is found guilty of a crime he did not commit despite the lack of evidence. The white racist jurors commit testimonial injustice because he is black, and this leads to his conviction. Fricker argues that social imagination unconsciously feeds into this judgement. It would be comforting to think that the underlying attitudes and assumptions of the jurors, in this case, would have changed significantly by the time of Fricker's writing—but as numerous cases that boosted the rise of the Black Lives Matter movement have demonstrated, such assumptions still affect the judgements of professionals and members of the public, causing them, in some cases, to dismiss the claims of a man being choked to death that he cannot breathe. Despite this being an unreliable and conflicting stereotype, it is a dominant stereotype nonetheless that causes serious harm.^58^ We owe it to ourselves and vulnerable children to reform for the better by listening, hearing, and creating a new language to finally put all sinister epistemic injustices to bed.

No doubt, some commentators will see this conclusion as doing no more than pointing out the obvious or will use the very fact that we could cite some literature on the topic as evidence that the debate is already happening. But if it were indeed obvious to all then the serious problems noted in this paper would simply no longer exist—what we need now is a focused debate that builds on the papers cited here with the explicit goal of changing our thinking and practice.

So, the observations and suggestions made here should be seen as an attempt to open up an explicit conversation about the issue, to develop an inclusive dialogue. We need to think critically about the language we use, our interpretations of the statements and behaviour of others, and the attitudes and assumptions that underlie them. This has always been an important way to overcome prejudice and improve our understanding of our fellow human beings, whatever their sex, race, social background, or age.

## CONFLICTS OF INTEREST

The authors declare no conflicts of interest.

## Data Availability

The data that support the findings of this study are from publicly available sources that are clearly referenced in the article.

## Footnotes

1 We are aware that this claim may seem incongruous, even bizarre, in the context of a paper that uses these labels throughout. We wanted this paper to be understood by and accessible to other members of the care community, and at the time of writing, the best way to be clear is to use the dominant terminology, which is also used in many of the important papers we have referenced in the article. However, we look forward to the day when others may look at this paper and react with the thought: “How could they use those labels?!” We do not assume to speak finally or authoritatively on the best alternative language to employ but invite readers to take this point on board as one of the challenges we face to improve our practices in future.

2 That is to say, this would be instantly recognized *today*. As the work of several of the authors cited in this paper reminds us, it took a long time for that recognition to become a feature of mainstream discourse. Even now, members of campaigning groups such as Black Lives Matter have had to remind the broader society that such recognition is by no means universal. The struggle for epistemic justice is, of course, an on‐going enterprise, but it is worth reminding ourselves that progress can be made.
